# Microbiological surveillance after automated reprocessing of gastrointestinal endoscopes in routine clinical practice: A prospective randomized microbiological surveillance study

**DOI:** 10.1371/journal.pone.0360048

**Published:** 2026-10-06

**Authors:** Masayuki Kato, Hideki Hayashi, Nobuharu Hironaka, Masafumi Chiba, Takahiro Abe, Nana Shimamoto, Mamoru Ito, Masakuni Kobayashi, Kazuki Sumiyama

**Affiliations:** 1 Department of Endoscopy, The Jikei University School of Medicine, Tokyo, Japan; 2 Kaigen Pharma Co., Ltd., Osaka, Japan; 3 IHI Logistics & Machinery Corporation, Tokyo, Japan; University of Nebraska Medical Center College of Medicine, UNITED STATES OF AMERICA

## Abstract

Microbiological surveillance is used to monitor the quality of gastrointestinal endoscope reprocessing, but prospective data comparing automated endoscope reprocessor systems under routine workflow conditions remain limited. We conducted an exploratory, single-center, prospective randomized surveillance study of 222 endoscope-use episodes involving patient-used forward-viewing upper and lower gastrointestinal endoscopes. Endoscope-use episodes were allocated 1:1:1 to strongly acidic electrolyzed water, ozonated water, or peracetic acid using an Excel-generated random-number procedure (n = 74 each). Assigned reprocessing was performed after standardized bedside pre-cleaning and manual cleaning. Post-reprocessing samples were obtained from predefined external and internal sites. Surveillance success was defined as ≤20 colony-forming units per sample at all sampled sites and absence of predefined indicator organisms. Success was achieved in 67/74 (90.5%) strongly acidic electrolyzed-water episodes, 71/74 (95.9%) ozonated-water episodes, and 71/74 (95.9%) peracetic-acid episodes. Risk differences in surveillance success versus peracetic acid were −5.4 percentage points (95% confidence interval, −14.6 to 3.2) and 0.0 percentage points (95% confidence interval, −7.7 to 7.7), respectively. In exploratory post hoc non-inferiority analyses using a −5 percentage-point margin, non-inferiority was not demonstrated for either functional-water system. A −10 percentage-point margin supported ozonated water but not strongly acidic electrolyzed water. Post hoc scope-level sensitivity analyses were directionally consistent with the primary analysis and highlighted the influence of repeated failures in an individual physical endoscope. These findings should be interpreted as exploratory microbiological quality-assurance data, not as evidence of clinical infection risk or comparative clinical effectiveness.

Study registration: University Hospital Medical Information Network Clinical Trials Registry (UMIN-CTR), UMIN000061627 (registered May 19, 2026; enrollment began November 8, 2024).

## Introduction

Flexible gastrointestinal endoscopes are semi-critical medical devices that contact mucous membranes and can be exposed to blood, intestinal contents, and other potentially infectious material during routine clinical use. Inadequate cleaning, disinfection, drying, or storage has been associated with healthcare-associated infections and outbreak events, particularly when device complexity or workflow deviations compromise reprocessing [1–5]. Reliable, reproducible reprocessing is therefore a central component of infection prevention in gastrointestinal endoscopy units.

Current guidance emphasizes a standardized multistep workflow that includes bedside pre-cleaning, meticulous manual cleaning with detergent flushing and channel brushing, high-level disinfection, rinsing, drying, and appropriate storage [1,2]. Peracetic acid (PAA)-based automated endoscope reprocessors (AERs) are widely used because PAA has broad antimicrobial activity under validated conditions. However, real-world microbiological performance depends on the entire reprocessing chain, including manual cleaning, endoscope design, AER cycle execution, staff adherence, drying, storage, and sampling sensitivity.

Microbiological surveillance provides a practical quality-assurance approach for assessing residual bioburden after reprocessing. Surveillance programs use quantitative culture thresholds and predefined indicator organisms to identify possible process deviations; they do not directly estimate patient-level infection incidence or prove absolute reprocessing safety [6–9].

Functional-water AER systems, including strongly acidic electrolyzed water and ozonated water, have been introduced in some clinical settings. These systems differ in generation method, active antimicrobial species, stability, delivery to internal channels, and process-control mechanisms. Prior clinical and experimental studies have evaluated electrolyzed acid water for endoscope reprocessing [10,11]. Importantly, a recent multicenter study in otorhinolaryngological endoscopy reported that electrolyzed acid water substantially reduced bacterial contamination after clinical use and supported its feasibility for less complex flexible endoscopes [12]. Whether such findings can be generalized to gastrointestinal endoscopes remains uncertain because gastrointestinal endoscopes have different contamination burdens, longer and more complex channels, and distinct clinical workflows.

Accordingly, we conducted an exploratory prospective randomized surveillance study to evaluate microbiological quality-assurance outcomes after reprocessing patient-used gastrointestinal endoscopes with two functional-water systems and a PAA-based system under routine clinical workflow conditions. The objective was not to demonstrate clinical infection prevention or device interchangeability, but to provide transparent, endoscope-level surveillance data that could inform quality-management programs and future confirmatory studies.

## Materials and methods

### Study design, ethics, and registration

This exploratory prospective randomized surveillance study was conducted at a single tertiary-care endoscopy center under routine clinical workflow conditions. The Institutional Review Board of The Jikei University School of Medicine approved the study protocol (No. 36–199; 12308). Enrollment began on November 8, 2024. The study was registered in the University Hospital Medical Information Network Clinical Trials Registry (UMIN-CTR; UMIN000061627) on May 19, 2026 and publicly released on May 20, 2026; the registration was therefore retrospective. All patients received an oral explanation of the study from a physician at the bedside before participation. A nurse was present during the consent process and documented the verbal consent in the electronic medical record. This oral consent and medical-record documentation procedure was approved by the Institutional Review Board. The study complied with the ethical principles of the Declaration of Helsinki.

The unit of allocation was the endoscope-use episode. Allocation was based on the scheduled examination list available on the preceding day, and the assigned reprocessing method was applied after completion of the clinical examination and standardized manual cleaning. The allocated intervention was an endoscope reprocessing method applied to the patient-used endoscope in that episode, and the outcome was a microbiological surveillance result rather than a patient health outcome. Therefore, the study is reported as a prospective randomized microbiological surveillance study rather than as a clinical trial designed to assess patient outcomes.

### Endoscopes and reprocessing workflow

Consecutive endoscope-use episodes involving patient-used forward-viewing upper and lower gastrointestinal endoscopes were included. Endoscope type and procedural factors, including biopsy, therapeutic intervention, and known viral infection status, were recorded from endoscopy reports and medical records. Diagnostic examination only was defined as an examination without biopsy or therapeutic intervention.

Eligible units were endoscope-use episodes involving patient-used flexible gastrointestinal endoscopes used for routine gastrointestinal endoscopic examination or treatment. Exclusion criteria were: (1) patients with prion disease and endoscopes used in such patients; (2) minors and endoscopes used in minors; (3) patients who declined study participation and endoscopes used in those patients; (4) endoscopes with leakage detected during the pre-disinfection leak test; and (5) cases considered inappropriate for inclusion by the principal investigator or study investigators.

After each examination, bedside pre-cleaning was performed immediately, followed by standardized manual cleaning with detergent flushing and channel brushing according to guidance for flexible gastrointestinal endoscope reprocessing [1,2]. Endoscope-use episodes were allocated to the three AER systems in a 1:1:1 ratio using the computer-generated random-number procedure specified in the study protocol. Using the list of scheduled endoscopic examinations available on the preceding day, random numbers were generated with the RAND function in Microsoft Excel. The list was sorted in descending order of these random numbers and divided into three parts: the first third was assigned to KD-1, the second third to OED, and the final third to PAA. After manual cleaning, each eligible endoscope underwent automated reprocessing with its assigned AER system. Personnel performing reprocessing and sample collection were not blinded to allocation. The external microbiological laboratory was not provided with AER allocation information and performed microbiological testing without knowledge of the assigned reprocessing system.

The three AER systems were KD-1 (Kaigen Pharma Co., Ltd., Osaka, Japan), which uses strongly acidic electrolyzed water; OED (IHI Logistics & Machinery Corporation, Tokyo, Japan), which uses ozonated water; and OER-6 (Olympus Corporation, Tokyo, Japan), a PAA-based system. Each AER was operated according to the manufacturer’s validated cycle conditions. Cycle parameters were not experimentally modified, preserving a pragmatic routine-practice comparison.

### Microbiological sampling and culture

Microbiological sampling was performed at two time points: immediately after completion of the examination before bedside pre-cleaning, and immediately after completion of the AER cycle. Post-reprocessing samples were collected before alcohol flushing, drying, or storage. For surface sampling, a microbiological swab moistened with sterile saline was used to wipe the entire insertion portion (site D) or the entire control section (site E) 10 times; a fixed surface area in cm^2^ was therefore not specified. For channel sampling (site F), 20 mL of sterile saline was flushed through the biopsy port, and the recovered fluid was collected at the distal forceps-channel outlet and submitted for microbiological culture. No neutralizing agent was used in the sampling or transport medium.

Samples were processed by an external microbiology laboratory (Japan Microbiology Clinic Co., Ltd., Tokyo, Japan). Quantitative aerobic bacterial culture was performed using soybean-casein digest (SCD) agar, with incubation at 35 °C for 48 h. Colony-forming units (CFU) were counted visually and reported as CFU per sample. Organisms were identified by 16S rDNA sequence analysis. Culture results were recorded separately according to sampling method, sampled site, and time point. Raw culture reports included exact CFU counts and categorical results such as “below detection limit” or “ < 20 CFU.” For endpoint classification, all results reported below the laboratory reporting threshold were classified as below the predefined CFU failure threshold. For descriptive numeric summaries and figures, results below the reporting threshold were coded as 0 CFU, while the original raw reporting categories were retained in the shared dataset.

### Outcomes

The primary outcome was microbiological surveillance success for each endoscope-use episode after automated reprocessing. Success was defined as both residual bacterial load ≤20 CFU per sample at every sampled site and absence of predefined indicator organisms after reprocessing. Indicator organisms were predefined in the study protocol as *Escherichia coli*, enterococci, members of the Enterobacteriaceae, *Pseudomonas aeruginosa* and other gram-negative nonfermenters, *Staphylococcus aureus*, and *Staphylococcus epidermidis*. Detection of any predefined indicator organism was classified as surveillance failure irrespective of the CFU count. Secondary outcomes included the distribution of CFU counts, failure components, and the distribution of failures according to endoscope type, sampling method, sampled site, procedural factor, individual endoscope identifier, and available repair history.

### Sample size and statistical analysis

Because robust preliminary data were unavailable for surveillance-based microbiological outcomes comparing these AER systems under routine workflow conditions, the sample size of 74 endoscope-use episodes per group was determined by feasibility during the predefined study period and the expected number of eligible routine endoscopic procedures at our institution. The study was therefore not powered for a definitive non-inferiority conclusion. The study protocol specified a non-inferiority comparison of microbiological surveillance success between each functional-water system and the PAA-based system but did not specify a numerical non-inferiority margin. Therefore, the −5 percentage-point margin used in the present analysis was post hoc. A −10 percentage-point margin was additionally examined as an exploratory sensitivity analysis. Accordingly, inferences based on both numerical margins were considered exploratory.

All evaluable endoscope-use episodes were included in the full analysis set. Surveillance success rates were summarized as proportions. Comparisons with the PAA-based group were expressed as risk differences in surveillance success with 95% confidence intervals calculated using the Newcombe method. Fisher’s exact test was performed descriptively. Subgroup and scope-level analyses were considered exploratory and were not adjusted for multiplicity because of the small number of surveillance failures. To address non-independence arising from repeated use of the same physical endoscope, post hoc sensitivity analyses were performed. A nonparametric cluster bootstrap used the anonymized physical-endoscope identifier as the resampling unit, retaining all endoscope-use episodes belonging to each resampled scope; 100,000 bootstrap replicates were used with random-number seed 20260812. In addition, a logistic generalized estimating equation model was fitted with physical endoscope as the clustering variable and an exchangeable working correlation structure. A further post hoc sensitivity analysis excluded all observations contributed by SCOPE005. These analyses were considered hypothesis-generating. Analyses were conducted using R (R Foundation for Statistical Computing, Vienna, Austria); the revised statistical code reproduces the primary and sensitivity analyses and records the R session information.

## Results

### Study cohort and procedural characteristics

A total of 222 eligible endoscope-use episodes were randomized, with 74 allocated to each system. All 222 completed the assigned reprocessing procedure and microbiological sampling and were included in the analysis. A separate prospective screening log of endoscope-use episodes excluded before randomization was not maintained, so the total numbers screened and excluded before randomization could not be reconstructed reliably. The distribution of upper and lower gastrointestinal endoscope-use episodes was comparable across groups: 45 upper and 29 lower episodes in the strongly acidic electrolyzed-water group, 46 upper and 28 lower episodes in the ozonated-water group, and 45 upper and 29 lower episodes in the PAA-based group (Table 1). Sampling was completed for all predefined sites in all analyzed episodes.

**Table 1:** Characteristics of endoscopes and procedural factors included in the study.

Characteristic	Strongly acidic electrolyzed water (n = 74)	Ozonated water (n = 74)	Peracetic acid (n = 74)
Upper GI endoscope-use episodes, n (%)	45 (60.8)	46 (62.2)	45 (60.8)
Lower GI endoscope-use episodes, n (%)	29 (39.2)	28 (37.8)	29 (39.2)
Age, years, mean ± SD	63.6 ± 11.0	62.1 ± 13.7	64.4 ± 11.9
Male sex, n (%)	41 (55.4)	47 (63.5)	47 (63.5)
Diagnostic examination only, n (%)	50 (67.6)	49 (66.2)	49 (66.2)
Biopsy performed, n (%)	18 (24.3)	14 (18.9)	16 (21.6)
Therapeutic intervention, n (%)	6 (8.1)	11 (14.9)	10 (13.5)
Cold polypectomy, n	5	10	8
Endoscopic mucosal resection, n	2	3	3
Esophageal dilation, n	0	0	1
Known viral infection, n (%)	3 (4.1)	2 (2.7)	2 (2.7)
All predefined sites sampled, n (%)	74 (100)	74 (100)	74 (100)

Diagnostic examination only was defined as an examination without biopsy or therapeutic intervention. Biopsy and therapeutic intervention were not mutually exclusive; one PAA-group examination included both and was counted in both categories. Therapeutic interventions included cold polypectomy, endoscopic mucosal resection, and esophageal dilation. Because more than one therapeutic procedure could be performed in the same examination, the sum of individual therapeutic procedures may exceed the number of examinations with therapeutic intervention. Known viral infection included documented viral infection markers recorded in the medical chart.

Mean age was 63.6 ± 11.0 years in the strongly acidic electrolyzed-water group, 62.1 ± 13.7 years in the ozonated-water group, and 64.4 ± 11.9 years in the PAA-based group; male sex accounted for 41/74 (55.4%), 47/74 (63.5%), and 47/74 (63.5%), respectively (Table 1). Detailed diagnostic categories were not systematically collected for comparative analysis.

Diagnostic examination only accounted for 50 (67.6%), 49 (66.2%), and 49 (66.2%) examinations, respectively. Biopsy was performed in 18 (24.3%), 14 (18.9%), and 16 (21.6%) examinations, and therapeutic intervention was performed in 6 (8.1%), 11 (14.9%), and 10 (13.5%) examinations, respectively. Biopsy and therapeutic intervention were not mutually exclusive; one PAA-group examination included both and was counted in both categories.

### Microbiological surveillance success

Surveillance success was achieved in 67 of 74 endoscope-use episodes (90.5%) in the strongly acidic electrolyzed-water group, 71 of 74 (95.9%) in the ozonated-water group, and 71 of 74 (95.9%) in the PAA-based group (Table 2). Compared with PAA, the risk difference in surveillance success was −5.4 percentage points (95% CI, −14.6 to 3.2) for strongly acidic electrolyzed water and 0.0 percentage points (95% CI, −7.7 to 7.7) for ozonated water. Fisher’s exact P values were 0.327 and 1.000, respectively. Using the post hoc −5 percentage-point margin, non-inferiority was not demonstrated for either functional-water system. Under the exploratory −10 percentage-point margin, ozonated water, but not strongly acidic electrolyzed water, met the margin.

**Table 2:** Surveillance success, failure components, and exploratory non-inferiority analysis.

Outcome	Strongly acidic electrolyzed water (n = 74)	Ozonated water (n = 74)	Peracetic acid (n = 74)
Surveillance success, n (%)	67 (90.5)	71 (95.9)	71 (95.9)
Surveillance failure, n (%)	7 (9.5)	3 (4.1)	3 (4.1)
Failure with CFU > 20, n	4	2	2
Failure with CFU ≤ 20 but indicator organism detected, n	3	1	1
Failure in upper GI endoscope-use episodes, n	0	2	0
Failure in lower GI endoscope-use episodes, n	7	1	3
Failure detected by swab sampling, n	6	3	2
Failure detected by flush-collect sampling, n	1	0	1
RD in surveillance success vs PAA, percentage points (95% CI)	−5.4 (−14.6 to 3.2)	0.0 (−7.7 to 7.7)	Reference
Post hoc NI margin −5 percentage points	Not demonstrated	Not demonstrated	--
Post hoc NI margin −10 percentage points	Not demonstrated	Supported	--
Fisher exact P value vs PAA	0.327	1.000	Reference

Surveillance success was defined as ≤20 colony-forming units per sample at all sampled sites and absence of predefined indicator organisms after reprocessing. Cases categorized as colony-forming units >20 may also have included detection of predefined indicator organisms. CFU, colony-forming units; PAA, peracetic acid.

Risk differences in surveillance success relative to PAA are shown in Fig 1.

**Fig 1:**
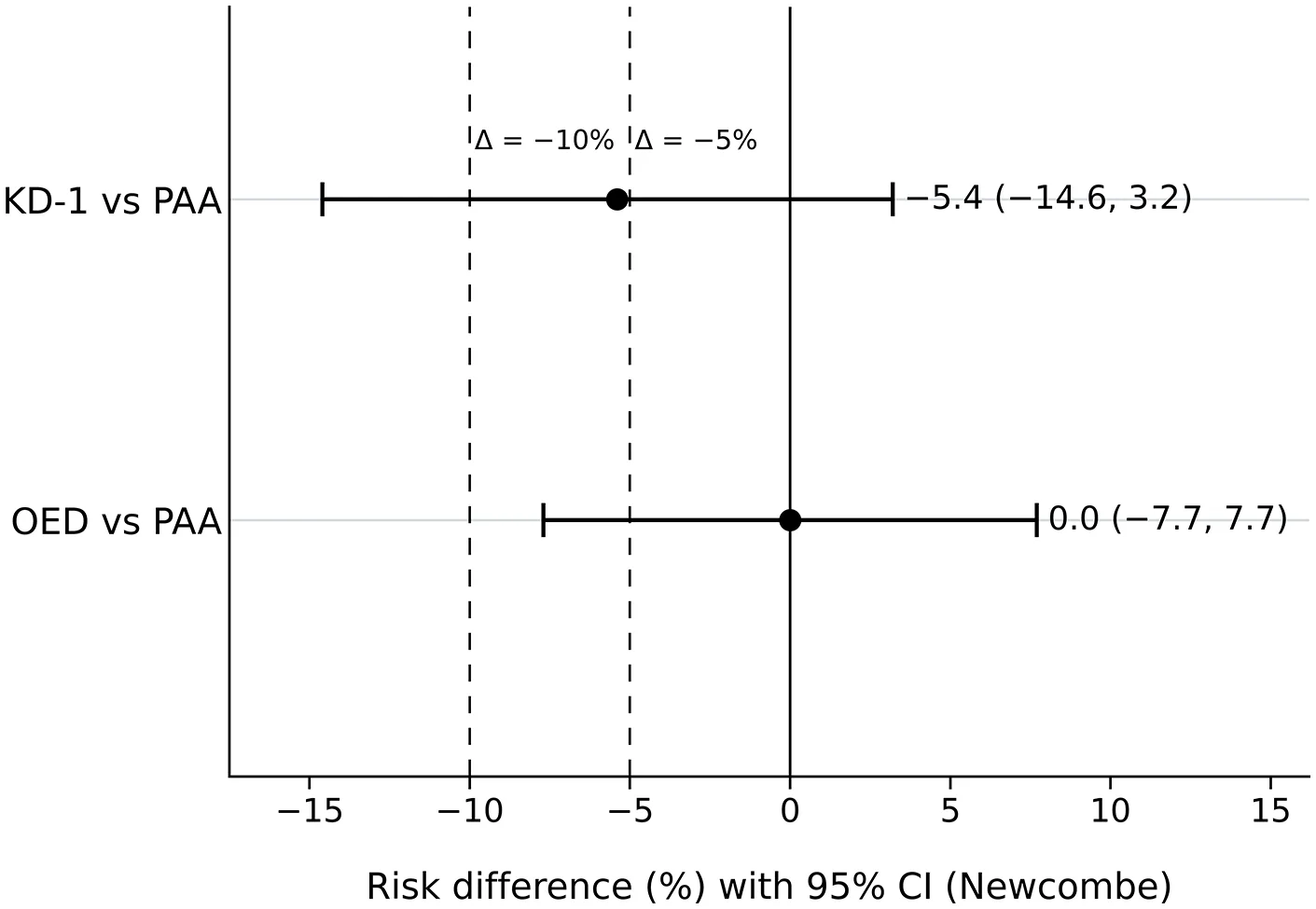
Risk differences in surveillance success relative to the peracetic acid group. Risk differences are shown with 95% confidence intervals calculated using the Newcombe method. Vertical reference lines indicate 0 percentage points and the post hoc exploratory non-inferiority margins of −5 and −10 percentage points. PAA, peracetic acid; CI, confidence interval; KD-1, automated endoscope reprocessor using strongly acidic electrolyzed water; OED, automated endoscope reprocessor using ozonated water.

In the post hoc physical-scope cluster bootstrap (100,000 replicates; seed 20260812), the 95% confidence interval was −16.4 to 3.7 percentage points for KD-1 versus PAA and −6.4 to 6.3 percentage points for OED versus PAA. In the post hoc logistic GEE analysis, the estimated exchangeable working correlation was 0.020; the marginal risk difference was −5.3 percentage points (95% CI, −15.2 to 4.7) for KD-1 versus PAA and +0.1 percentage points (95% CI, −6.2 to 6.4) for OED versus PAA. These scope-level results are presented as hypothesis-generating sensitivity analyses (S1 Fig).

The distributions of CFU counts before and after reprocessing, pooled across sampling sites, are shown in Fig 2.

**Fig 2:**
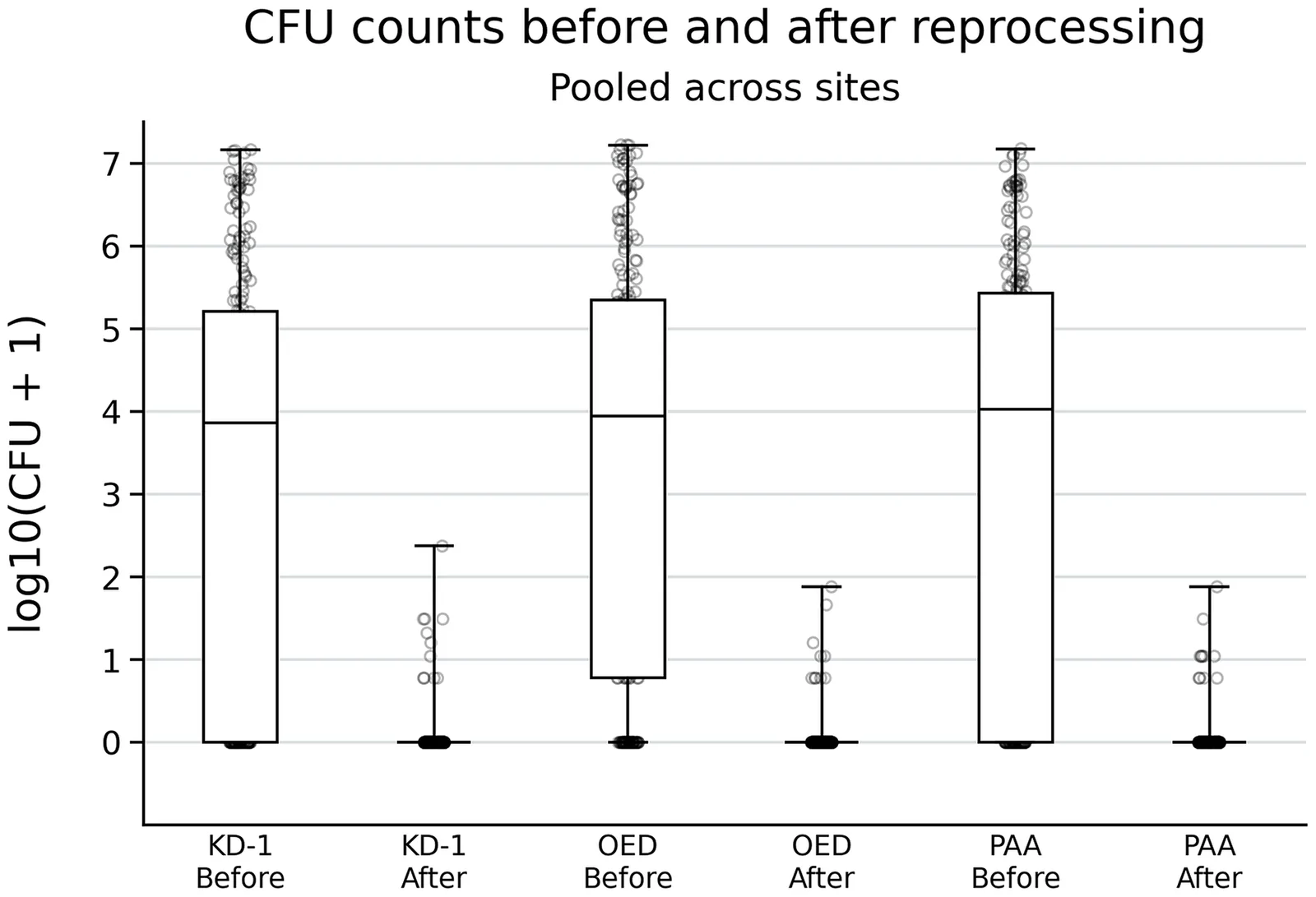
Distribution of colony-forming unit counts before and after reprocessing. Values are pooled across sampling sites and displayed on a log10(CFU + 1) scale. Points represent individual samples. Boxes indicate the interquartile range, central lines indicate the median, and whiskers extend to the minimum and maximum values. CFU, colony-forming units; KD-1, automated endoscope reprocessor using strongly acidic electrolyzed water; OED, automated endoscope reprocessor using ozonated water; PAA, peracetic acid. For visualization, values reported below the microbiological reporting threshold were plotted as 0; this transformation was used only for graphical presentation and did not alter classification of surveillance success or failure.

### Failure patterns and scope-level findings

Thirteen endoscope-use episodes failed to meet the predefined surveillance criteria after reprocessing. Eight failures had > 20 CFU per sample; seven of these also contained a predefined indicator organism. Five additional failures had ≤ 20 CFU per sample but contained a predefined indicator organism. Predefined indicator organisms were detected in 7 KD-1, 3 OED, and 2 PAA episodes. Because the composite endpoint was assessed per endoscope-use episode, an episode was counted only once as a surveillance failure even when more than one failure criterion was met. Eleven failures occurred in lower gastrointestinal endoscopes and two in upper gastrointestinal endoscopes. Eleven were detected by swab sampling and two by flush-collect sampling.

Failures were observed predominantly in lower gastrointestinal endoscopes (11 of 86 lower endoscope-use episodes) and in 2 of 136 upper endoscope-use episodes. Because several failures clustered in a single lower gastrointestinal endoscope, these findings were interpreted descriptively and were not used to infer independent differences in reprocessing performance by endoscope type.

The 222 endoscope-use episodes were contributed by 43 unique physical endoscopes, with a median of 4 included uses per scope (IQR, 3–8; range, 1–13). The numbers of distinct scopes contributing observations to the KD-1, OED, and PAA groups were 28, 30, and 31, respectively. Eighteen scopes contributed observations to all three AER groups, 10 contributed to two groups, and 15 contributed to only one group; thus, 28 of 43 scopes contributed observations to more than one AER group. SCOPE005 (PCF-H290ZI) contributed six episodes: three KD-1 episodes, all of which failed, one OED episode that succeeded, and two PAA episodes that succeeded. In a post hoc analysis excluding all six SCOPE005 observations, surveillance success was 67/71 (94.4%) for KD-1, 70/73 (95.9%) for OED, and 69/72 (95.8%) for PAA. The risk difference was −1.5 percentage points (95% CI, −9.9 to 6.7; P = 0.719) for KD-1 versus PAA and +0.1 percentage points (95% CI, −7.7 to 7.9; P = 1.000) for OED versus PAA. Repair-history data were available for 189 of 222 episodes and missing for 33. Failures occurred in 11/150 (7.3%) episodes involving endoscopes with two or more prior repairs and 1/39 (2.6%) involving endoscopes with zero or one prior repair; one additional failure occurred among episodes with missing repair-history data.

In exploratory analyses, failures were not significantly associated with biopsy, therapeutic intervention, cold polypectomy, endoscopic mucosal resection, esophageal dilation, or known viral infection status. Before reprocessing, detected microorganisms reflected oral, enteric, and environmental flora. After reprocessing, organisms detected in surveillance failures included enteric, environmental, and skin-associated organisms.

## Discussion

In this exploratory prospective randomized surveillance study conducted under routine clinical workflow conditions, most forward-viewing gastrointestinal endoscopes met predefined microbiological criteria after automated reprocessing across all three systems. Surveillance success was 90.5% with KD-1 and 95.9% with both OED and PAA. In exploratory post hoc analyses, non-inferiority was not demonstrated for either functional-water system using a −5 percentage-point margin. Using a −10 percentage-point margin, OED but not KD-1 met the margin. These results are best interpreted as microbiological quality-assurance data rather than as evidence of clinical infection risk, patient-level safety, or definitive device equivalence.

A key strength of this study is that it extends prior clinical evidence on electrolyzed acid water to the setting of patient-used gastrointestinal endoscopes. Okano et al. reported that electrolyzed acid water was feasible for disinfecting flexible otorhinolaryngological endoscopes in a multicenter clinical setting and also evaluated in vitro antimicrobial activity [12]. Their study provided important real-world evidence for less complex flexible endoscopes. The present study addresses a related but distinct question by evaluating gastrointestinal endoscopes, which have longer channels, different contamination burdens, and more complex reprocessing workflows. The findings should therefore be viewed as complementary evidence supporting device- and setting-specific microbiological surveillance rather than as simple extrapolation across endoscope categories.

The distinction between microbiological surveillance and patient-level safety assessment is critical. An approximately 95% surveillance success rate should not be interpreted as meaning that approximately 5% of reprocessed endoscopes were clinically unsafe. The endpoint was not sterilization, post-procedure infection, or direct patient harm, but achievement of predefined microbiological criteria intended for quality assurance [6,7]. The PAA-based system, used as the routine-practice comparator, also showed occasional surveillance failures, supporting the interpretation that such findings may reflect sampling stringency, residual environmental bioburden, scope-level factors, and workflow variability rather than insufficient intrinsic disinfectant efficacy alone.

The failure components support this interpretation. Eight unsuccessful episodes exceeded the colony-count threshold, whereas five had colony counts within the threshold but failed because a predefined indicator organism was detected. *Staphylococcus epidermidis* was retained as an indicator organism because it was explicitly included in the pre-enrollment study protocol, consistent with earlier ESGE-ESGENA surveillance concepts that included staphylococci among indicator organisms. However, low-level recovery of coagulase-negative staphylococci may also represent skin or environmental contamination; the clinical significance of an isolated low-level finding should therefore be interpreted cautiously. Such findings should prompt process review, repeat sampling, and scope-level monitoring rather than immediate inference of clinical infection risk.

The results also illustrate the limitations of applying a non-inferiority framework to high-success reprocessing outcomes in a feasibility-based study. A non-inferiority margin should be specified and justified at the design stage [13]. The protocol specified a non-inferiority comparison but did not prespecify a numerical margin; consequently, the −5 and −10 percentage-point margins used in the present analyses are post hoc and exploratory. The confidence intervals were not sufficiently narrow to establish non-inferiority at −5 percentage points for either functional-water system. The −10 percentage-point analysis supported OED but not KD-1. Scope-level sensitivity analyses were added post hoc and should likewise be considered hypothesis-generating rather than confirmatory.

Strongly acidic electrolyzed water and ozonated water should not be considered interchangeable simply because both are categorized as functional water. These systems differ in generation method, active antimicrobial species, stability, delivery to internal channels, and process-control mechanisms. Mechanistic disinfectant performance also depends on exposure time, organic load, microbial species, and resistance characteristics [14]. These considerations support device-specific validation and surveillance rather than class-level assumptions.

Several failures occurred in lower gastrointestinal endoscope-use episodes and clustered in specific physical scopes. Excluding SCOPE005 attenuated the KD-1 versus PAA point estimate from −5.4 to −1.5 percentage points, indicating that this individual physical endoscope contributed importantly to the observed group-level difference; nevertheless, the lower confidence bound remained below the post hoc −5 percentage-point margin. The cluster-bootstrap confidence interval was wider than the endoscope-use-level Newcombe interval for KD-1 (−16.4 to 3.7 vs −14.6 to 3.2) but slightly narrower for OED (−6.4 to 6.3 vs −7.7 to 7.7). Because the intervals were generated using different estimation procedures, cluster resampling does not necessarily produce uniformly wider confidence intervals. The greater widening for KD-1 is consistent with concentration of three failures in repeated KD-1 uses of SCOPE005, whereas the three OED failures occurred in three different physical endoscopes and the GEE working correlation was low (0.020). The slightly narrower OED cluster-bootstrap interval should not be interpreted as evidence of greater efficacy or inherently greater precision. The repair-history analysis also suggested a higher numerical failure frequency among endoscopes with two or more prior repairs, but the small number of failures, missing repair-history data, endoscope type, cumulative use, and repeated observations limit causal interpretation. These findings are hypothesis-generating and support future longitudinal scope-level surveillance.

This study has limitations. First, it was conducted at a single center and used a feasibility-based sample size; it was not powered for a definitive non-inferiority conclusion, and the numerical non-inferiority margins were not prespecified. Second, it included forward-viewing upper and lower gastrointestinal endoscopes but not duodenoscopes or echoendoscopes, which have more complex structures and have been associated with contamination and multidrug-resistant organism transmission [3–5,15,16]. Third, detailed patient-level factors such as *Helicobacter pylori* infection status, recent antibiotic exposure, proton pump inhibitor use, diabetes, malignancy, and chemotherapy were not comprehensively evaluated. Fourth, post-procedure infectious complications were not assessed. Fifth, the endpoint was based on aerobic bacterial culture and predefined indicator organisms and did not directly assess virucidal, mycobactericidal, or sporicidal activity. Although *Bacillus subtilis* was detected in one unsuccessful case, standardized spore-challenge testing was not performed; bench-based testing remains necessary for disinfectant-specific claims [1,2,14,17,18]. Formal cost-effectiveness analysis was also beyond the scope of this study. Sixth, raw culture reports included categorical values below the laboratory reporting threshold; these were handled conservatively as below the predefined CFU failure threshold and coded as 0 CFU only for descriptive numeric summaries, with raw categories preserved in the shared dataset. Seventh, because individual physical endoscopes were reused during the study, endoscope-use episodes were not fully independent. Although the primary analysis was performed at the endoscope-use level as originally planned, the post hoc scope-cluster bootstrap and GEE sensitivity analyses were added to quantify the effect of within-scope correlation and should be interpreted as hypothesis-generating. Eighth, no neutralizing agent was used in the sampling or transport medium. Residual disinfectant carried over into post-reprocessing samples may therefore have affected microbial recovery. Because the residual characteristics of peracetic acid, strongly acidic electrolyzed water, and ozonated water differ, the magnitude of any carryover effect may also have differed among the three systems; differential residual-disinfectant carryover cannot be excluded as a potential source of measurement bias.

## Conclusions

In this prospective randomized microbiological surveillance study, most forward-viewing gastrointestinal endoscopes met predefined surveillance criteria after automated reprocessing across all three systems under routine clinical workflow conditions. Using a post hoc −5 percentage-point margin, non-inferiority was not demonstrated for either functional-water system. Because the numerical non-inferiority margin was not prespecified, the study was feasibility-based, and scope-level analyses were post hoc, these comparisons should be interpreted as exploratory. The findings support continued microbiological process monitoring and future longitudinal scope-level studies but should not be interpreted as evidence of clinical infection risk, patient-level safety, or comparative clinical effectiveness.

## Supporting information

S1 Dataset Anonymized endoscope-level dataset underlying the analyses. (CSV)

S2 Dataset Plot-ready sample-level CFU dataset underlying Fig 2. (CSV)

S3 File R code for the primary, physical-scope sensitivity, procedure-overlap, repair-history, and failure-component analyses reported in the manuscript. (R)

S4 Table English summary tables generated from the final source data workbook, including characteristics, CFU-threshold outcomes, risk/Fisher summaries, site-level CFU summaries, failure cases, organism detections, and the case-level dataset. (XLSX)

S1 Fig Post hoc scope-level sensitivity analyses of risk differences in microbiological surveillance success relative to the peracetic acid group. The cluster-bootstrap analysis resampled physical endoscopes as clusters and retained all endoscope-use episodes belonging to each resampled scope (100,000 replicates; seed 20260812). The GEE analysis used physical endoscope as the clustering variable with an exchangeable working correlation structure. These analyses were not prespecified and are presented as hypothesis-generating sensitivity analyses.
(TIFF)

S5 Table Protocol-defined surveillance-failure episodes after endpoint re-audit. This table lists the 13 endoscope-use episodes classified as failures after re-auditing all 222 observations against the complete pre-enrollment indicator-organism definition and identifies the applicable failure criterion or criteria.
(CSV)

S1 Text Anonymized English translation of the patient information sheet used in this study. (PDF)

S2 Text Anonymized English translation of the study protocol. (PDF)
