# Supplementary material for: Microbiological surveillance after automated reprocessing of gastrointestinal endoscopes in routine clinical practice: A prospective randomized microbiological surveillance study
Source: PLoS One. 2026 Oct 6;21(10):e0360048. doi: 10.1371/journal.pone.0360048 (PMC13641389; doi:10.1371/journal.pone.0360048)
Supplement: Supplementary file 1 [file pone.0360048.s007.pdf]

Information Document for Obtaining Consent

Study title
Study to Evaluate the Disinfection Efficacy of Functional Water for Gastrointestinal Endoscopes

Version 1.0 Prepared: June 13, 2024
Version 1.1 Prepared: July 10, 2024
Version 1.2 Prepared: August 19, 2024

Principal investigator
Department of Endoscopy, The Jikei University School of Medicine
[redacted]

1. Conduct of the research

<Introduction>

This information document has been prepared to ask for your cooperation in this study. Please read it carefully. Later, we will ask you orally whether you consent to participate. If you do not consent, you will not be disadvantaged in any way. If you have any questions or concerns, please feel free to ask the person explaining the study, your attending physician, or the contact listed in Section 15, Consultation contacts.

<What is clinical research?>

Progress and development in medicine are important for providing patients with more effective and safer diagnosis and treatment of their diseases. Many studies are needed to advance these diagnostic and treatment methods, and some must be conducted in humans (healthy individuals or patients). This is called "clinical research."

Clinical research is conducted in accordance with the Declaration of Helsinki (the World Medical Association's ethical principles for medical research involving human subjects) and the Ethical Guidelines for Life Science and Medical Research Involving Human Subjects (guidelines established primarily by the Ministry of Health, Labour and Welfare). Its appropriateness is reviewed from ethical and scientific perspectives by the Ethics Committee established at The Jikei University School of Medicine. Following approval, the research is conducted with permission from the director of the hospital where it takes place.

2. Name of the research institution and principal investigator

<Principal investigator>

Professor, Department of Endoscopy, The Jikei University School of Medicine
[redacted]

3. Purpose and significance of the research

The purpose of this study is to disinfect endoscopes used in patients with the approved endoscope washer-disinfectors "CLEANTOP KD-1" and "OED-1000S Plus" and to examine their disinfection efficacy.

4. Research methods and duration

<Research methods>

After the endoscopic examination, the endoscope used in the patient will be disinfected using an endoscope washer-disinfector. Feces, blood, and mucus adhering to the endoscope before and after disinfection will be collected as samples for bacterial testing. Patient information (year and month of birth, sex, comorbidities, etc.) will also be collected from medical records. No additional tests or medical examinations will be performed for this study.

<Expected duration of participation>

Your participation in this study will last approximately 30 minutes, during which the endoscopic examination is performed.

5. Reasons for selection as a research participant

<Inclusion criteria>

Patients attending the hospital for an endoscopic examination.

<Exclusion criteria>

(1) Patients infected with prion disease.
(2) Patients who are minors.

6. Anticipated risks and benefits

Because this study is performed on endoscopes that have already been used in patients, participation in this study entails no risks or disadvantages.

7. Freedom to withdraw consent

Even if you agree to participate in this study, you may withdraw at any time. To withdraw your consent, please telephone the contact listed in Section 15, Consultation contacts.

Please understand, however, that you cannot withdraw participation after the study findings have been published.

8. Freedom to participate

Your cooperation in this study is entirely voluntary. You will not be disadvantaged in any way if you do not agree to participate.

9. Public disclosure of information about the research

The findings of this study may be presented at academic conferences or published in scientific journals. Before publication, we will confirm that the information has been appropriately processed so that specific individuals cannot be identified and participants are not disadvantaged with respect to their privacy.

Please understand that any rights arising from the findings of this study belong to the investigators conducting it.

Information about the study and its progress will also be made publicly available in the following database for anyone to view.

University Hospital Medical Information Network Center: UMIN (URL: https://www.umin.ac.jp/)

10. How to obtain or view the study protocol and related materials

At your request, you may obtain or view materials describing the study plan and methods, to the extent that doing so does not risk compromising other participants' personal information or the originality of the research.

If you wish to view these materials, please contact the person listed in Section 15, Consultation contacts.

11. Handling of personal information and related information

<Handling of personal information>

Personal information about participants in this study will be handled in accordance with the Ethical Guidelines for Life Science and Medical Research Involving Human Subjects,

the institution's rules and procedures, the Act on the Protection of Personal Information, applicable ordinances, and other requirements.

Your personal information obtained for this study (including sensitive personal information such as diagnoses, medical history, and genetic information) will be handled strictly and appropriately to prevent disclosure or loss.

For requests to disclose your personal information, complaints, or inquiries, please contact the principal investigator or the personal information protection consultation desk listed in Section 15, Consultation contacts.

Information that directly identifies you will not be provided to collaborating research institutions or other parties.

<Processing information so that specific individuals cannot be identified>

When handling samples and information in this study, we will remove your personal information (such as your name, address, and hospital identification number) and replace it with unrelated symbols or numbers.

Consequently, when your samples and information are used for this study or exchanged with investigators at other institutions, they will not be identifiable as yours.

When unrelated symbols or numbers are assigned, a table or record linking them to your personal information (a table linking the collected samples and information to your name or other identifiers) will be created. This table or record will be strictly managed under the responsibility of the principal investigator at this institution.

12. Storage and disposal of samples and information

Samples such as blood and information provided by you will be stored under the management of the principal investigator and used for research after personal information has been removed.

Information in electronic form will be stored on a computer that is not connected to a network; paper records will be stored in a lockable cabinet, with full security measures in place.

They will be appropriately retained until the later of five years after the study ends or three years after the date on which the final publication of the study results is reported. Longer retention may be required under the rules of medical or academic research institutions.

For disposal, blood samples will be autoclaved. Data stored on electronic media will be erased, and the media will then be physically destroyed.

Samples and information provided by study participants, including you, will be collected at the institution below after personal information has been removed. Records of the provision and receipt of samples and information will be retained for three years after the study ends by the providing institution and for five years after the study ends by the receiving institution. Longer retention may be required under institutional rules.

Receiving institution: Japan Microbiology Clinic Co., Ltd.
Responsible person: Representative Director and President [redacted]
Samples/information: Feces, blood, and mucus adhering to flexible endoscopes
Procedure/method: Refrigerated courier service

13. Research funding and conflicts of interest

<Research funding>

The expenses of this study will be covered by research funds of the department/laboratory to which the principal investigator belongs.

<Conflicts of interest>

This study receives equipment on loan free of charge from Kaigen Pharma Co., Ltd. and IHI Logistics Systems Co., Ltd. The study will nevertheless be conducted with efforts to ensure transparency and fairness.

The investigators comply with the university's conflict-of-interest management rules and have completed the procedures required by the Conflict of Interest Management Committee.

14. Handling of research results

This item is not applicable because no tests or treatments are performed for research purposes. The findings will not directly concern your health or treatment, so they will not be explained to you individually.

15. Consultation contacts

If you wish to withdraw consent, have questions about this study, feel unwell, wish to request disclosure of personal information, or have other concerns, please feel free to contact your attending physician or the following investigators at any time.

Research institution/department: Department of Endoscopy, The Jikei University Hospital
Telephone: [redacted] (extension: [redacted])
Principal investigator: [redacted] ([redacted])
Study investigator: [redacted] ([redacted])

In addition to the principal investigator, the following contact handles requests for disclosure of personal information, complaints, and inquiries.

<Personal information protection consultation desk>

Main University Hospital [redacted]
Direct telephone: [redacted]
Office hours: 9:00 a.m. to 4:00 p.m., excluding days when the hospital is closed

16. Provision of samples or information to persons outside Japan

There are no plans to provide the information you supply to medical or academic research institutions outside Japan.

17. Financial burden or compensation

Participation in this study will not increase your costs beyond those of usual medical care. No payments, such as compensation to reduce the burden of participation, will be made.

18. Other treatment methods

This study involves people who are already scheduled for an endoscopic examination. Even if you do not participate, you will receive the same endoscopic care as you would if you participated.

19. Medical care after participation in the research

This item is not applicable because no new treatments or additional tests will be performed for this research.

20. Compensation for health-related harm

This study only observes usual treatment and examinations, so no health-related harm resulting from the study is anticipated.

21. Possibility that your information or samples may be used in other research, and how to obtain information about such use

If a purpose for using samples or information that was not specified when consent was obtained is subsequently identified, a study protocol will be prepared or amended and undergo ethical review. Participants will then be notified of the newly specified purpose, or the information will be published on the university hospital website (https://jikei.bvits.com/rinri/publish.aspx) or by other means. Participants will be guaranteed an opportunity to withdraw consent to that research being conducted.

22. Access to your information

This study will not undergo checks to determine whether it is being conducted properly (called monitoring or audits). Therefore, persons who are not involved in this study will not view your information.

End
