# Supplementary material for: Microbiological surveillance after automated reprocessing of gastrointestinal endoscopes in routine clinical practice: A prospective randomized microbiological surveillance study
Source: PLoS One. 2026 Oct 6;21(10):e0360048. doi: 10.1371/journal.pone.0360048 (PMC13641389; doi:10.1371/journal.pone.0360048)
Supplement: Supplementary file 2 [file pone.0360048.s008.pdf]

English translation - identifying details redacted

Study Protocol

Study title
Study to Evaluate the Disinfection Efficacy of Functional Water for Gastrointestinal Endoscopes

Version 1.0 Prepared: May 21, 2024
Version 1.1 Prepared: June 13, 2024
Version 1.2 Prepared: July 10, 2024
Version 1.3 Prepared: August 19, 2024

Principal investigator:
Name: [redacted]
Research institution, department, and position:
The Jikei University School of Medicine
Department of Endoscopy, Professor

Contents

1. Research organization and implementation structure 3
2. Purpose and significance of the research 3
3. Research methods 4
4. Target sample size and planned study period 7
5. Selection of research participants 7
6. Scientific rationale for the research 8
7. Procedures for obtaining informed consent 8
8. Handling of personal information and related information 10
9. Anticipated risks and benefits 10
10. Storage and disposal of samples and information 11
11. Reports to the head of the research institution: content and methods 12
12. Research funding and conflicts of interest 12
13. Public disclosure of research information 12
14. Handling of research results 13
15. Responding to inquiries from participants and related persons 13
16. Procedures for obtaining informed consent from representatives 13
17. Procedures for obtaining informed assent 13
18. Research in an urgent and evident life-threatening situation 13
19. Financial burden and compensation for participants 13
20. Response to serious adverse events (invasive research) 14
21. Compensation for health-related harm (invasive research) 14
22. Medical care after the study (interventional research) 14
23. Outsourced work and supervision of contractors 14
24. Secondary use of samples and information obtained from participants ... 14
25. Monitoring and audit arrangements and procedures
(research involving invasiveness and intervention) 14
26. References 15

1) Research organization and implementation structure

1.1. Principal investigator

Professor, Department of Endoscopy, The Jikei University School of Medicine [redacted]
Role: Responsible for preparing the protocol, data management, and overall study supervision.

1.2. Co-investigators within the university

Associate Professor, Department of Endoscopy, The Jikei University School of Medicine [redacted]
Role: Protocol preparation, data collection, obtaining participant consent, responsibility for allocation and analysis, and manuscript preparation.
Assistant Professor, Department of Endoscopy, The Jikei University School of Medicine [redacted]
Role: Data collection and obtaining participant consent.
Assistant Professor, Department of Endoscopy, The Jikei University School of Medicine [redacted]
Role: Data collection and obtaining participant consent.
Assistant Professor, Department of Endoscopy, The Jikei University School of Medicine [redacted]
Role: Data collection and obtaining participant consent.
Graduate Student, Department of Endoscopy, The Jikei University School of Medicine [redacted]
Role: Data collection and obtaining participant consent.

2) Purpose and significance of the research

2.1. Purpose

This study will be conducted to confirm that the disinfection efficacy of the CLEANTOP KD-1 flexible endoscope washer-disinfector (hereafter, test device (1)) and the OED-1000S Plus ozonated-water endoscope disinfector (hereafter, test device (2)) for gastrointestinal flexible endoscopes after clinical use is equivalent to that of the OER-6 endoscope washer-disinfector (hereafter, the control device). The significance of this study is that it can demonstrate that cleaning gastrointestinal flexible endoscopes using the test devices enables the provision of safe medical care.

2.2. Background and significance

Flexible endoscopes are used in a broad range of clinical fields, including gastroenterology, urology, and otorhinolaryngology, for both diagnosis and various treatments. Endoscopes may contact mucous membranes and non-intact skin. Under the Spaulding classification, widely used to determine cleaning, disinfection, and sterilization procedures, they are classified as semicritical devices requiring high-level disinfection (elimination of all microorganisms except when large numbers of spores are present).

Guidelines and manuals on cleaning and disinfecting flexible endoscopes have been published in gastroenterology [1)], urology [2)], respiratory medicine [3)], otorhinolaryngology [4)], and other fields. They list peracetic acid, glutaraldehyde, ortho-phthalaldehyde, and functional water (strongly acidic electrolyzed water and ozonated water) as disinfectants.

Strongly acidic electrolyzed water is obtained at the positive electrode by electrolyzing an aqueous sodium chloride solution and has a broad antimicrobial spectrum. Its safety has been demonstrated in biological safety tests, and experimental studies have shown lower cytotoxicity than peracetic acid and chlorine dioxide preparations [5-7)]. Ozonated water is produced by dissolving ozone (O3), generated by applying energy such as high voltage to oxygen (O2), in water. Disinfection against various bacteria, fungi, and viruses, and viral inactivation, have been

reported. A major characteristic is its environmental compatibility, as it decomposes rapidly and ultimately returns to oxygen (O2) [8-11].

Strongly acidic electrolyzed water has been shown to disinfect gastrointestinal and otorhinolaryngological endoscopes after clinical use [12,13]. Urata et al. [14] likewise demonstrated the efficacy of ozonated water for disinfecting gastrointestinal endoscopes. However, no study has yet established non-inferiority to peracetic acid, a widely used high-level disinfectant.

3) Research methods

3.1. Study design

This is a prospective cross-sectional observational study involving bacterial culture testing before and after cleaning and disinfection with test devices (1) and (2) and the control device. It will examine gastrointestinal flexible endoscopes used for observation, diagnosis, imaging, and treatment of internal lumens and cavities, the oral cavity, and the trachea (hereafter, the study endoscopes).

3.2. Study outline

Co-investigators will perform the endoscopic examinations. Samples will be collected from the study endoscopes before and after cleaning and disinfection ((1) the entire portion inserted into the patient (insertion section), (2) the entire portion operated by the examiner (control section), and (3) the channel) for bacterial testing to evaluate disinfection efficacy. As in usual practice, the sterilization service contractor will clean and disinfect the endoscopes used in patients with test device (1), test device (2), or the control device.

3.2.1. Study schema

Consent obtained

↓

Eligibility confirmed

↓

Target sample size
Test device (1): 74 cases
Test device (2): 74 cases
Control device: 74 cases
Endoscope enrollment: Ethics approval to March 31, 2025
Study period: Ethics approval to June 30, 2025

↓

Samples collected from gastrointestinal flexible endoscopes after clinical use, before and after cleaning/disinfection, for bacterial testing.
Test devices (1) and (2) and the control device are used for cleaning/disinfection.

↓

Timing of efficacy evaluation: After completion of culture testing

3.2.2. Sampling from study endoscopes (before cleaning and disinfection)

Samples will be collected before manual cleaning. Sampling will be performed at three sites: (1)-1, the entire portion inserted into the patient (insertion section); (2)-1, the entire portion operated by the examiner (control section); and (3)-1, the channel. For (1)-1 and (2)-1, a swab intended for bacterial testing will be moistened with sterile physiological saline, and the sampling site will be wiped 10 times. The swab will be placed in a sterile tube and stored at room temperature until all samples have been collected. For (3)-1, 20 mL of sterile physiological saline will be instilled through the biopsy port and recovered into a sterile tube at the distal forceps outlet, then stored at room temperature as for (1)-1 and (2)-1.

3.2.3. Cleaning and disinfection using the test and control devices

Manual cleaning will follow the hospital's cleaning and disinfection manual. Allocation will then be performed by simple randomization. Cleaning and disinfection with the test or control device will follow Appendices 1-4 and the hospital's cleaning and disinfection manual.

For allocation by simple randomization, numbers will be assigned using Excel's RAND function to the list of scheduled endoscopic examinations on the preceding day. The list will be sorted in descending numerical order and divided into three parts. The top third will undergo cleaning and disinfection with test device (1), the next third with test device (2), and the remaining third with the control device.

3.2.4. Sampling from study endoscopes (after cleaning and disinfection)

Samples will be collected after disinfection with the test or control device. Sampling will be performed at three sites: (1)-2, the entire portion inserted into the patient (insertion section); (2)-2, the entire portion operated by the examiner (control section); and (3)-2, the channel. For (1)-2 and (2)-2, a swab intended for bacterial testing will be moistened with sterile physiological saline, and the sampling site will be wiped 10 times. The swab will be placed in a sterile tube and stored at room temperature until all samples have been collected. For (3)-2, 20 mL of sterile physiological saline will be instilled through the biopsy port and recovered into a sterile tube at the distal forceps outlet, then stored at room temperature as for (1)-2 and (2)-2.

After collection, samples will be refrigerated (2-8 degrees C), and all samples will be shipped together to the receiving laboratory under refrigerated conditions on the same day.

After disinfection with the test device and sampling, usual cleaning and disinfection will be performed according to the institution's manual to ensure that the test device does not affect patients.

3.2.5. Culture testing of collected samples

Culture testing will be outsourced to Japan Microbiology Clinic. After culture testing, samples will not be stored but will be promptly discarded by Japan Microbiology Clinic.
Sample destination (Japan Microbiology Clinic)
Japan Microbiology Clinic Co., Ltd.
[redacted]
TEL: [redacted] FAX: [redacted]
Office hours: Weekdays, 9:00 a.m. to 7:00 p.m.

3.3. Endpoints

The primary endpoint is a between-group assessment of non-inferiority in the proportion of study endoscopes for which disinfection by the test or control device is judged effective

(the proportion with effective disinfection).

Based on the ESGE-ESGENA Guideline for Quality Assurance in Reprocessing: Microbiological Surveillance Testing in Endoscopy [15)], effective disinfection is defined as a post-disinfection bacterial count of no more than 20 CFU per sampling site and no detection of indicator organisms.

Proportion with effective disinfection = Number of study endoscopes judged effectively disinfected / Number of study endoscopes included in the analysis (analysis population defined in 11.1.2).
Indicator organisms: Escherichia coli, enterococci, and Enterobacteriaceae
: Pseudomonas aeruginosa and other gram-negative nonfermenters
: Staphylococcus aureus, Staphylococcus epidermidis

If bacteria are detected on a study endoscope, identification and tabulation of bacterial species will be a secondary endpoint.

3.4. Observations and tests

Collected samples will undergo culture testing. Samples contain feces, blood, and mucus.
The microorganisms examined will be aerobic bacteria. If bacteria are detected, counts will be quantified and species identified.
Patient characteristics (year and month of birth, sex, examination date, comorbidities, and presence or absence of prion disease infection) will also be tabulated.

3.5. Data management

Data will be managed in Excel. Each patient will be assigned a study ID. Information that could identify the patient to persons outside the participating medical institution (name, address, telephone number, medical record number, etc.) will not be recorded.

3.6. Statistical analysis

3.6.1. Full Analysis Set (FAS)

The FAS will comprise study endoscopes used in patients enrolled in this study. Gastrointestinal flexible endoscopes found to involve major protocol violations (violation of enrollment criteria, refusal to participate, non-enrollment, or other major violations of study procedures) will be excluded.

3.6.2. Per Protocol Set (PPS)

The PPS is defined as enrolled study endoscopes with more than 20 CFU detected at at least one sampling site before disinfection and without major protocol violations, such as violation of enrollment criteria (violation of enrollment criteria, refusal to participate, non-enrollment, or other major violations of study procedures).

3.6.3. Primary analysis

The PPS will be analyzed, and one-sided 95% confidence intervals will be estimated for each group. Fisher's exact test will also be used to confirm non-inferiority of the proportion with effective disinfection for the test devices versus the control device. The significance level will be one-sided 5%.

If bacteria are detected after disinfection with a test or control device, the relationship between the detected species and the species before disinfection will be examined descriptively.

4) Target sample size and planned study period

4.1. Target sample size

Target sample size: Test device (1): 74 cases
Test device (2): 74 cases
Control device: 74 cases
The target sample size is 222 cases across the three groups.

4.2. Rationale for the target sample size

Considering the report by Okano et al. [13)] and feasibility, the target sample size was set as follows. Assuming a proportion with effective disinfection of 99%, a confidence level of 95%, and a confidence interval width no greater than 5%, the target sample size n is calculated as follows:

n = (2 x 1.96 x sqrt(0.99 x (1 - 0.99)) x (1 / 0.05))^2 = 60.85

Therefore, at least 61 cases are required.
In the report by Okano et al. [13)], approximately 17% of endoscopes had no bacteria detected before disinfection. Taking such cases into account, the target was set at 74 cases per group.

4.3. Planned study period

Study endoscope enrollment period: Date of ethics approval to March 31, 2025
Study implementation period: Date of ethics approval to June 30, 2025

5) Selection of research participants

5.1. Inclusion criteria

5.1.1. Inclusion criteria

Gastrointestinal flexible endoscopes used for observation, diagnosis, imaging, and treatment of internal lumens and cavities, the oral cavity, and the trachea in patients attending for an endoscopic examination will be eligible.

5.1.2. Rationale for the inclusion criteria

Because this study examines disinfection efficacy, all patients and endoscopes will be eligible unless an exclusion criterion applies.

5.2. Exclusion criteria

5.2.1. Exclusion criteria

(1) Patients infected with prion disease and gastrointestinal flexible endoscopes used in such patients.
(2) Patients who are minors and gastrointestinal flexible endoscopes used in such patients.
(3) Patients who refuse participation and gastrointestinal flexible endoscopes used in such patients.
(4) Leakage detected in the study endoscope by a leak test before disinfection.
(5) Cases considered unsuitable for this study by the principal investigator or a co-investigator.

5.2.2. Rationale for the exclusion criteria

(1) Prion inactivation is known to require special treatment; such cases are therefore excluded [16].
(2)-(5) These are excluded for case registration.

6) Scientific rationale for the research

6.1. Rationale for the study design

Strongly acidic electrolyzed water has been shown to disinfect gastrointestinal and otorhinolaryngological endoscopes after clinical use [12,13]. Urata et al. [14] likewise demonstrated the efficacy of ozonated water for disinfecting gastrointestinal endoscopes. However, no study has yet established non-inferiority to peracetic acid, a widely used high-level disinfectant. This study will contribute to establishing methods for cleaning and disinfecting gastrointestinal flexible endoscopes and providing safe medical care.

6.2. Appropriateness of the study implementation structure

To conduct this study properly and safely, the principal investigator will endeavor to secure a sufficient number of medical personnel (co-investigators, sterilization contractors, etc.) and use appropriate facilities.

6.3. Appropriateness of risks and benefits for participants

Disinfection in this study is performed on devices already used in patients, and no burden from participation is anticipated. Likewise, participation provides no special benefit to individual patients.

7) Procedures for obtaining informed consent

7.1. Ethical considerations

7.1.1. Regulatory compliance

This study will comply with the Declaration of Helsinki and the Ethical Guidelines for Life Science and Medical Research Involving Human Subjects (Ministry of Education, Culture, Sports, Science and Technology; Ministry of Health, Labour and Welfare; and Ministry of Economy, Trade and Industry; partially amended March 27, 2023).

7.1.2. Compliance with the study protocol

Investigators participating in this study will comply with this protocol unless doing so compromises patient safety or human rights.

7.1.3. Deviations from the study protocol

Investigators must not deviate from or amend the protocol before obtaining permission from the head of the research institution based on prior review by the Ethics Committee.

When the principal investigator becomes aware of a deviation or change, the details and reasons, together with proposed amendments to the protocol or related documents if necessary, will be promptly submitted to the Ethics Committee and reported to the Committee and the head of the research institution.

All protocol deviations will be documented by the principal investigator, including their reasons and the measures taken.

7.2. Method of informed consent

This study falls into the following categories.

1.	☐ Invasive (including minimally invasive)<br>☑ Non-invasive
2.	☐ Interventional<br>☑ Non-interventional
3.	☑ (1) Uses samples and information newly obtained for research purposes<br>☐ (2) Uses only information newly obtained for research purposes<br>☐ (3) Uses existing samples and information<br>☐ (4) Uses existing information only
4.	☑ (1) Samples/information are exchanged with other institutions<br>☐ (2) No samples/information are exchanged with other institutions

Informed consent will be obtained in accordance with Chapter 4, Section 8, 1(1)(b)(ii)(1) of the Ethical Guidelines for Life Science and Medical Research Involving Human Subjects. Because this study involves many cases and obtaining written consent is impractical, the study will be conducted after oral informed consent has been obtained.

Investigators will explain the items stipulated by the Ethical Guidelines for Life Science and Medical Research Involving Human Subjects to participants and record the method and content of the explanation and the consent obtained in Appendix 5, Case Registration Form/Case Report Form. If information that could affect consent becomes available, or changes to the study plan that could affect consent are made, investigators will promptly provide the information to participants, ascertain in advance whether they wish to participate, and obtain renewed consent in accordance with the above method.

The following matters will be explained using language that is easy for participants to understand.
1. Conduct of the research
2. Name of the research institution and principal investigator
3. Purpose and significance of the research
4. Research methods and duration
5. Reasons for selection as a research participant
6. Anticipated risks and benefits
7. Freedom to withdraw consent
8. Freedom to participate
9. Public disclosure of information about the research
10. How to obtain or view the study protocol and related materials
11. Handling of personal information and related information
12. Storage and disposal of samples and information
13. Research funding and conflicts of interest
14. Handling of research results
15. Consultation contacts
16. Provision of samples or information to persons outside Japan

17. Financial burden or compensation
18. Other treatment methods
19. Medical care after participation in the research
20. Compensation for health-related harm
21. Possibility that your information or samples may be used in other research, and how to obtain information about such use
22. Access to your information

8) Handling of personal information and related information

8.1. Handling of personal information and related information

Investigators and the head of the research institution will comply with the Ethical Guidelines for Life Science and Medical Research Involving Human Subjects, institutional rules and procedures, the Act on the Protection of Personal Information, applicable ordinances, and other requirements when handling personal information, anonymized information, pseudonymized information, and personal-related information.

Investigators will appropriately handle personal information and related information obtained through the research and held by their institution to prevent leakage, loss, or damage and otherwise ensure secure management.

The principal investigator, in cooperation with the head of the institution, will provide the necessary guidance and supervision to other investigators handling such information to ensure its appropriate handling during the study.

When publishing results, investigators will confirm that information has been appropriately processed so that specific individuals cannot be identified and participants do not suffer disadvantages relating to privacy.

Information that identifies individual participants in this study will not be shared with other research institutions or other parties.

8.2. Methods of processing information so that individuals cannot be identified

Investigators will remove personal information, including names, initials, and patient IDs, from the samples and information collected in this study and assign new research IDs or numbers (hereafter, study IDs) so that specific individuals cannot be identified.

During this processing, a table or record linking personal information to study IDs (hereafter, the linkage table) will be created so that information processed to prevent identification (pseudonymized information) can be linked back to personal information.

The linkage table will be used when necessary, such as when a participant withdraws consent or refuses participation, to link pseudonymized information back to personal information and discard that participant's samples or information. Under the principal investigator's responsibility, the linkage table will be stored in a locked cabinet within the institution, on a protected server or cloud service, or on a password-protected computer.

Publications of this study's results will not include information identifying individual participants.

9) Anticipated risks and benefits

9.1. Anticipated benefits

Disinfection in this study is performed on devices already used in patients, so participation provides no special benefit to individual patients.

9.2. Anticipated burdens and disadvantages

Disinfection in this study is performed on devices already used in patients, so no burdens or disadvantages from participation are anticipated.

9.3. Anticipated risks

Disinfection in this study is performed on devices already used in patients. After disinfection with the test device and sampling, usual cleaning and disinfection will be performed according to the institution's manual. Therefore, no risks from participation are anticipated.

9.4. Methods to minimize risks

Because no risks are anticipated, no discontinuation criteria are specified for individual cases.

10) Storage and disposal of samples and information

10.1. Storage of samples

10.1.1. Definition of samples to be stored

Samples consist of feces, blood, and mucus adhering to flexible endoscopes.

10.1.2. Method of sample storage

Samples will be refrigerated (2-8 degrees C).

10.1.3. Sample storage period

All samples will be shipped together to Japan Microbiology Clinic under refrigerated conditions on the same day.

10.1.4. Method of sample disposal

After culture testing, Japan Microbiology Clinic will promptly discard the samples.

10.2. Storage of information

10.2.1. Definition of information to be stored

Copies of applications and reports; notifications from the Ethics Committee and the head of the research institution; the linkage table; copies of Case Registration Forms/Case Report Forms; records of the provision of samples/information; and other documents or records necessary to ensure data reliability, including medical records.

10.2.2. Information storage methods, retention period, and disposal

In principle, the principal investigator will retain the source materials defined in 10.2.1 until the later of five years after the study ends or three years after the date on which the final publication of the study results is reported. If other retention rules applicable at the study institution require a longer period, those rules will be followed. Electronic records will be stored by the data manager on a password-protected standalone computer not connected to the institution's LAN, and paper records will be stored in a lockable cabinet in the departmental office.

When records are discarded after the retention period, computer data will be erased using erasure software, and paper records will be shredded with due care for personal information.

10.3. Transfer of samples and information between institutions

Because this study involves transfers of samples and information between institutions, transfers between the providing and receiving institutions will follow Annex 2, "Creation and retention of records of transfers of samples and information between institutions."

11) Reports to the head of the research institution: content and methods

The principal investigator will report information about this study in writing to the head of the participating medical institution in accordance with the following provisions.
1) Before conducting the research (including research conducted under an amended protocol; the same applies below), the principal investigator will prepare a protocol, obtain Ethics Committee approval, and obtain permission from the heads of the research institution and the institution conducting the research.
2) If the principal investigator obtains facts or information that compromise or may compromise the study's ethical appropriateness or scientific rationale and are considered likely to affect its continuation, these will be reported without delay to the heads of the institution and the institution conducting the research. The study will be suspended or discontinued, or the protocol amended, as necessary.
3) If the principal investigator obtains facts or information that compromise or may compromise the appropriateness of study conduct or the reliability of results, these will be promptly reported to the heads of the research institution and the institution conducting the research. The study will be suspended or discontinued, or the protocol amended, as necessary.
4) In principle, the principal investigator will report annually to the head of the research institution on study progress, adverse events associated with study conduct, and storage and management of samples and information obtained from human subjects.
5) When the study ends (including discontinuation; the same applies below), the principal investigator will report to the heads of the research institution and the institution conducting the research.

12) Research funding and conflicts of interest

12.1. Research funding

The expenses of this study will be covered by research funds of the department/laboratory to which the principal investigator belongs.

12.2. Conflicts of interest and personal income

This study receives equipment on loan free of charge from Kaigen Pharma Co., Ltd. and IHI Logistics Systems Co., Ltd. The study will nevertheless be conducted with efforts to ensure transparency and fairness.

The investigators comply with the institution's conflict-of-interest management rules and have completed the procedures required by the committee managing conflicts of interest.

13) Public disclosure of research information

An outline and other information about this study will be registered in the following public database before the study begins, updated as appropriate following protocol amendments and study progress, and the results registered without delay when the study ends.

Database for registration: University Hospital Medical Information Network Center (UMIN)
When the study ends, its results will be disclosed without delay through conference presentations, journal publications, or other means, after taking measures necessary to protect the human rights of participants and related persons and the rights and interests of investigators and related persons.

14) Handling of research results

This item is not applicable because no tests or treatments are performed for research purposes. The findings are not expected to relate directly to participants' health or treatment and will therefore, in principle, not be explained individually.

15) Responding to inquiries from participants and related persons

Questions and inquiries about this study may be directed to:
Research institution/department: Department of Endoscopy, The Jikei University School of Medicine
Contact person: [redacted]
Telephone (extension): [redacted] ([redacted])

16) Procedures for obtaining informed consent from representatives

16.1. Participants requiring consent from a representative

This item is not applicable because this study does not include participants who require informed consent from a representative.

17) Procedures for obtaining informed assent

This item is not applicable because school-age minors are not included as participants.

18) Research in an urgent and evident life-threatening situation

This study does not involve research conducted in an urgent and evident life-threatening situation.

19) Financial burden and compensation for participants

19.1. Financial burden on participants

No financial burden will arise for individual patients during study participation.

19.2. Compensation for participants

No payments to reduce the burden of medical expenses, travel expenses, or similar expenses during study participation will be made.

20) Response to serious adverse events (invasive research)

This item is not applicable because the study is not invasive.

21) Compensation for health-related harm (invasive research)

This item is not applicable because the study involves procedures and sampling from medical devices after clinical use, and no health-related harm attributable to the study is anticipated.

22) Medical care after the study (interventional research)

This item is not applicable because the study involves procedures and sampling from medical devices after clinical use. Patients will receive usual medical care.

23) Outsourced work and supervision of contractors (when work is outsourced)

Culture testing of collected samples will be outsourced to Japan Microbiology Clinic Co., Ltd. The microorganisms examined will be aerobic bacteria. If bacteria are detected, counts will be quantified and species identified.
Japan Microbiology Clinic Co., Ltd.
[redacted]
TEL: [redacted] FAX: [redacted]
Office hours: Weekdays, 9:00 a.m. to 7:00 p.m.

24) Supervision method: The contractor will be required to report the status of the work to the investigators. Secondary use of samples and information obtained from participants

If a purpose for using samples or information that was not specified when consent was obtained is subsequently identified, a study protocol will be prepared or amended and undergo ethical review. Participants will then be notified of the newly specified purpose, or the information will be published on the university hospital website (https://jikei.bvits.com/rinri/publish.aspx) or by other means. Participants will be guaranteed an opportunity to withdraw consent to that research being conducted.

25) Monitoring and audit arrangements and procedures (research involving invasiveness and intervention)

25.1. Monitoring

No monitoring will be performed because this study is non-invasive and non-interventional.

25.2. Audits

No audits will be performed because this study is non-invasive and non-interventional.

26) References

26.1. Literature

1) Iwakiri R, Tanaka K, Gotoda T, Oka S, et al. Guidelines for standardization of cleaning and disinfection of gastrointestinal endoscopes. Gastroenterological Endoscopy. 2018;60(7):1370-1396.
2) Japanese Urological Association, Committee for the Development of Guidelines for Infection Control in Urology, et al. Guidelines for infection control in urology. Japanese Journal of Urology. 2009;100(4):1-27.
3) Japan Society for Respiratory Endoscopy, Safety Committee. Guidelines for infection control in urology. Journal of the Japan Society for Respiratory Endoscopy. 2017;39(6):p.Tebiki4.
4) Suzuki K, Omori K, Saito K, et al. Guide to infection control for otorhinolaryngological endoscopes. Journal of the Oto-Rhino-Laryngological Society of Japan. 2016;119(6):916-925.
5) Iwasawa A. Biological characteristics of electrolyzed acidic water (hypochlorous acid water). Journal of Japanese Society for Dialysis Therapy. 2015;48(2):90-93.
6) Iwasawa A, Nakamura R. Cytotoxicity of antiseptics: In vitro and in vivo comparison of strongly acidic electrolyzed water, povidone-iodine, chlorhexidine gluconate, and benzalkonium chloride preparations. Journal of the Japanese Association for Infectious Diseases. 2003;77(5):316-322.
7) Iwasawa A, Nakamura R. Antimicrobial effects and cytotoxicity of commercially available oxidizing agents. Japanese Journal of Environmental Infections. 2000;15(2):183-189.
8) Naito S. Ozonated-water generators and microbial control using ozonated water. 1. Types and characteristics of ozonated-water generators. Journal of Antibacterial and Antifungal Agents, Japan. 1998;26(12):713-724.
9) Yamasaki K, Nakamuro K. Inactivation of norovirus using low-concentration ozonated water. Journal of Antibacterial and Antifungal Agents, Japan. 2012;40(4):199-204.
10) Nakamuro K, Nakata H, Yamasaki K, et al. Method for evaluating the inactivation effect of low-concentration ozonated water against novel influenza virus. Journal of Antibacterial and Antifungal Agents, Japan. 2012;40(6):485-491.
11) Kamase Y. Endoscope sterilizer using ozone. Journal of the Institute of Electrostatics Japan. 2011;35(4):167-171.
12) Tsuji S, Kawano S, Oshita M, et al. Endoscope Disinfection Using Acidic Electrolytic Water. Endoscopy. 1999;31(7):528-535.
13) Okano T, Sakamoto T, Ishikawa S, Sakamoto S, Mizuta M, et al. Disinfection of otorhinolaryngological endoscopes with electrolyzed acid water: A cross-sectional and multicenter study. PLoS ONE. 2022;17(10):e0275488.
14) Urata M, Isomoto H, Kohno S, et al. Comparison of the Microbicidal Activities of Superoxidized and Ozonated Water in the Disinfection of Endoscopes. The Journal of International Medical Research. 2003;31:299-306.
15) Beilenhoff U, Neumann C, Rey J, et al. ESGE-ESGENA Guideline for Quality Assurance in Reprocessing: Microbiological Surveillance Testing in Endoscopy. Endoscopy. 2007;39(2):175-81.
16) Guidelines for the Prevention of Prion Disease Infection (2020 edition). http://prion.umin.jp/guideline/cjd_2020.pdf

26.2. Appendices

Appendix 1: CLEANTOP KD-1 package insert
Appendix 2: OED-1000S Plus package insert
Appendix 3: CLEANTOP KD-1 operating protocol

Appendix 4: OED-1000S Plus operating protocol
Appendix 5: Case Registration Form/Case Report Form
Appendix 6: Guide to completing and correcting Case Report Forms

Annex 1. Handling of personal information at The Jikei University School of Medicine

1-1. Processing to prevent identification of individuals	☑ Processing performed<br>☐ No processing<br>[Reason:]
1-2. Table linking personal information and study IDs (linkage table)	☑ A linkage table will be created<br>☐ No linkage table will be created<br>☐ A linkage table is created outside this university (only processed samples/information are handled here)<br>☐ Information is already processed when obtained (e.g., secondary use of previous research or an anonymous questionnaire)<br>☐ Other [Details:]
1-3. Linkage table manager (Personal information manager at this university for this study)	Department: Department of Endoscopy, The Jikei University School of Medicine<br>Name: [redacted]
1-4. Location of the linkage table	[redacted]
1-5. Personal information protection consultation contact	Principal investigator, and<br>Main University Hospital [redacted]<br>Direct telephone: [redacted]<br>Office hours: 9:00 a.m. to 4:00 p.m., excluding days when the hospital is closed

Annex 2. Creation and retention of records of transfers of samples and information between institutions

The creation and retention of records of transfers of samples and information handled in this study with collaborating research institutions, institutions that only provide samples/information, or research cooperation institutions are specified below. The list of collaborating institutions and their principal investigators, persons who only provide samples/information, research cooperation institutions, and their affiliated institutions is as specified in the study protocol.

Handling at the providing institution		Handling at the receiving institution	
(1) Samples/information provided	☑ As described in the study protocol<br>☐ Other [ ]	(12) Samples/information received	☑ Same as (1)<br>☐ Other [ ]
(2) How samples/information were obtained	☐ Obtained during medical care<br>☑ Obtained without payment after informed consent from the individual for use in this research	(13) How samples/information were obtained at the providing institution	☑ Same as (2)<br>☐ Other [ ]
(3) Individuals from whom provided samples/information originate	☐ As stated in the consent form<br>☑ Provided after pseudonymization	(14) Individuals from whom received samples/information originate	☑ The received samples/information themselves will be retained<br>☐ Other [ ]
(4) Timing of creation of provision records	☑ Each time provision occurs<br>☐ Compiled at continuing review or completion<br>☐ Other [ ]	(15) Timing of creation of receipt records	☑ Each time provision occurs<br>☐ Compiled at continuing review or completion<br>☐ Other [ ]
(5) Medium for provision records	☐ Electronic file on a PC<br>PC location [ ]<br>☐ Electronic storage medium such as DVD-ROM or USB memory<br>☑ Paper records<br>☐ Other [ ]	(16) Medium for receipt records	☐ Electronic file on a PC<br>PC location [ ]<br>☐ Electronic storage medium such as DVD-ROM or USB memory<br>☑ Paper records<br>☐ Other [ ]
(6) Creator of provision records	☑ Record creator at the providing institution:<br>Institution: The Jikei University School of Medicine<br>Department: Endoscopy<br>Name: [redacted]<br>Position: Associate Professor<br>☐ As listed in the attached list (if a separate list exists)<br>☐ Other [ ]	(17) Creator of receipt records	☑ Record creator at the receiving institution:<br>Institution: Japan Microbiology Clinic Co., Ltd.<br>Name: [redacted]<br>Position: Representative Director<br>☐ As described in the study protocol (if listed there)<br>☐ Other [ ]
(7) Documents substituting for provision records	☐ None<br>☐ Yes (MTA/DTA and consent forms substitute for transfer records)<br>☑ Yes (this protocol and consent forms substitute for transfer records)<br>☐ Yes (notification form for provision of samples/information to another institution substitutes for records)	(18) Availability of documents substituting for receipt records	☐ None<br>☐ Yes (MTA/DTA and consent forms substitute for transfer records)<br>☑ Yes (this protocol and consent forms substitute for transfer records)<br>☐ Yes (notification form for provision of samples/information to another institution substitutes for records)

	☐ EDC log records substitute for provision records<br>☐ Other [ ]		☐ EDC log records substitute for provision records<br>☐ Other [ ]
(8) Storage location of provision records	☑ Stored in [redacted] (records-management cabinet).<br>☐ Transfer logs recorded and stored on the receiving institution's EDC server<br>☐ Other [ ]	(19) Storage location of receipt records	☑ Stored in [redacted] (records-management cabinet).<br>☐ Transfer logs recorded and stored on the EDC server<br>☐ Other [ ]
(9) Person responsible for and location of linkage table management	☑ If the providing institution is this university: as described in Annex 1<br>☐ If the providing institution is another institution: as described in the study protocol<br>☐ Other [ ]	(20) Receiving institution and responsible person	☑ If the receiving institution is another institution:<br>Institution: Japan Microbiology Clinic Co., Ltd.<br>Name: [redacted]<br>Position: Representative Director<br>☐ If the providing institution is another institution: as described in the study protocol<br>☐ Other [ ]
(10) Receiving institution and responsible person	☑ If the receiving institution is another institution: as described in the study protocol<br>☐ Receiving institution and responsible person:<br>Institution [ ]<br>Department [ ]<br>Name [ ] Position [ ]	(21) Providing institution and responsible person	☑ If the providing institution is another institution: as described in the study protocol<br>☐ Providing institution and responsible person:<br>Institution [ ]<br>Department [ ]<br>Name [ ] Position [ ]
(11) Retention period for provision records	☑ Until three years have elapsed from the date of provision of (1). Longer retention may apply under institutional rules.<br>☐ Other [ ]	(22) Retention period for receipt records	☑ More than five years after completion of this study is reported. Longer retention may apply under institutional rules.<br>☐ Other [ ]
